# Antiepileptic activity and subtype-selective action of flupirtine at GABA_A_ receptors

**DOI:** 10.1186/2050-6511-13-S1-A90

**Published:** 2012-09-17

**Authors:** Mirnes Bajrić, Felicia Klinger, Ulla Schandl, Helmut Kubista, Stefan Boehm

**Affiliations:** 1 Department of Neurophysiology and Neuropharmacology, Center for Physiology and Pharmacology, Medical University of Vienna, 1090 Vienna, Austria

## Background

Flupirtine is used as analgesic drug with muscle-relaxant properties. In addition, it has been suggested to possess antiepileptic properties. Recently, flupirtine has been revealed to simultaneously act at K_V_7 channels and GABA_A_ receptors. Here, antiepileptic activity and underlying mechanisms of action of flupirtine were investigated.

## Methods

We used the patch clamp technique and primary cultures of hippocampal neurons or transfected tsA cells to investigate effects of flupirtine.

## Results

In hippocampal neurons, flupirtine reduced seizure-like activity with no effect at 1 to 3 µM, and maximal effects at 10 to 30 µM; it enhanced currents through K_V_7 channels with EC_50_ values at 6 µM. Flupirtine (30 µM) modulated GABA-induced currents in hippocampal neurons by reducing EC_50_ values for GABA threefold and maximal current amplitudes by 15%. Hence, flupirtine acted as an uncompetitive antagonist. Flupirtine did not alter rise time, decay time, or amplitudes of miniature inhibitory postsynaptic currents (mIPSCs), but enhanced the bicuculline-sensitive tonic current. When phasic GABAergic inhibition was blocked by penicillin G (5 mM), flupirtine enhanced maximal amplitudes of GABA-evoked currents by 43%, but hardly affected EC_50_ values. As these results suggested that flupirtine was able to differentiate between different GABA_A_ receptor subtypes, its effects on recombinant GABA_A_ receptors were investigated in tsA cells. With α1β2γ2 receptors, flupirtine reduced EC_50_ values for GABA threefold and maximal current amplitudes by 25%; with α1β2 receptors, it reduced EC_50_ values for GABA twofold, but reduced maximal current amplitudes by 35%.

## Conclusions

These results indicate that flupirtine (i) exerts antiepileptic activity, (ii) modulates tonic, but not phasic, GABAergic inhibition and blocks K_V_7 channels in hippocampal neurons, and (iii) affects GABA_A_ receptors in a subunit-dependent manner.

